# A Vertebral Artery Dissection with Basilar Artery Occlusion in a Child

**DOI:** 10.1155/2014/706147

**Published:** 2014-12-23

**Authors:** Katleen Devue, Annemie Van Ingelgem, Katrien De Keukeleire, Marc De Leeuw

**Affiliations:** ^1^Department of Emergency Medicine, ASZ Aalst, 9300 Aalst, Belgium; ^2^Department of Radiology, ASZ Aalst, 9300 Aalst, Belgium; ^3^Forensic Medicine, Forensic Pathology Department, Ghent University Hospital, 9000 Ghent, Belgium

## Abstract

This paper presents the case report of an 11-year-old boy with an acute dissection with thrombosis of the left vertebral artery and thrombosis of the basilar artery. The patient was treated with acute systemic thrombolysis, followed by intra-arterial thrombolysis, without any clinical improvement, showing left hemiplegia, bilateral clonus, hyperreflexia, and impaired consciousness. MRI indicated persistent thrombosis of the arteria basilaris with edema and ischemia of the right brainstem. Heparinization for 72 hours, followed by a two-week LMWH treatment and subsequent oral warfarin therapy, resulted in a lasting improvement of the symptoms. Vertebral artery dissection after minor trauma is rare in children. While acute basilar artery occlusion as a complication is even more infrequent, it is potentially fatal, which means that prompt diagnosis and treatment are imperative. The lack of class I recommendation guidelines for children regarding treatment of vertebral artery dissection and basilar artery occlusion means that initial and follow-up management both require a multidisciplinary approach to coordinate emergency, critical care, interventional radiology, and child neurology services.

## 1. Background

Firstly this paper highlights the importance of prompt diagnosis in a rare case where diagnosis can be a pitfall because the clinical presentation ranges from mild transient symptoms such as vertigo and headache to severe stroke with high morbidity. Secondly it clarifies the need for multidisciplinary approach regarding treatment because of a lack of class I recommendations for stroke in children. Coordination of emergency care, critical care, interventional radiology, and (child) neurology services is necessary in order to maximize the patient's chances of an improved outcome.

## 2. Case Presentation

**Figure 1:**
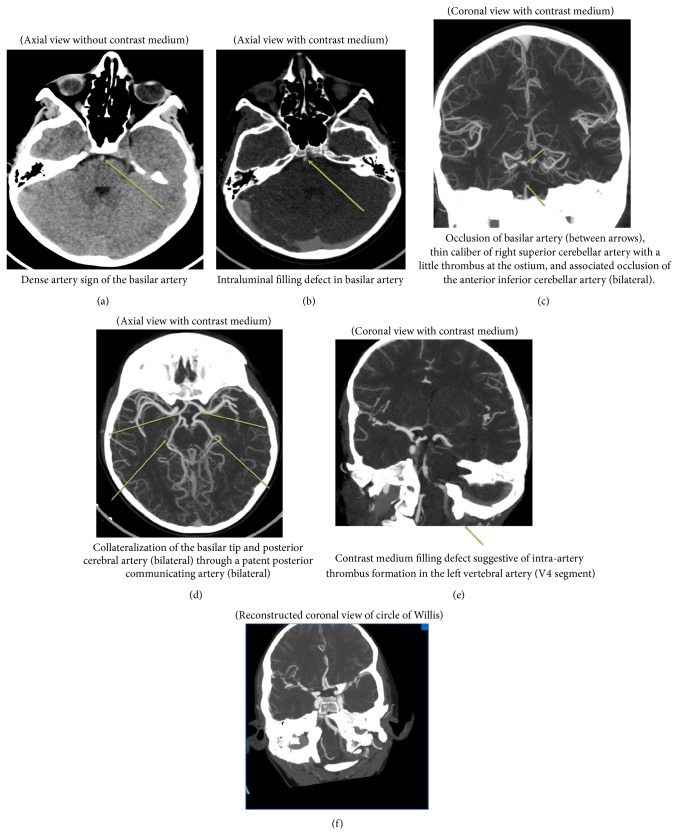
CAT and angioscan of the brain on admission.

An 11-year-old boy was brought by his mother to our emergency department, presenting symptoms including a sudden headache and dizziness after diving into a swimming pool. Upon his arrival at the emergency department the patient scored a PedNIHSS of 5. He was sleepy but arousable, with stable blood pressure of 131/78 mmHg and pulse of 96' min. He had a mydriatic right pupil, minor drift of his right arm, and mildly reduced sensibility of the right side of his body. According to his mother, the boy had fallen on his upper chest while inline skating a day earlier without any further complaints until he dove into the swimming pool. Blood was drawn, an intravenous access was placed, and a CAT scan of the brain and cervical vessels and angioscan of the brain were performed without delay. The scans revealed acute dissection of the boy's left vertebral artery with partial occlusion and thrombosis of the basilar artery (Figures 1(a)–1(f)).

**Figure 2:**
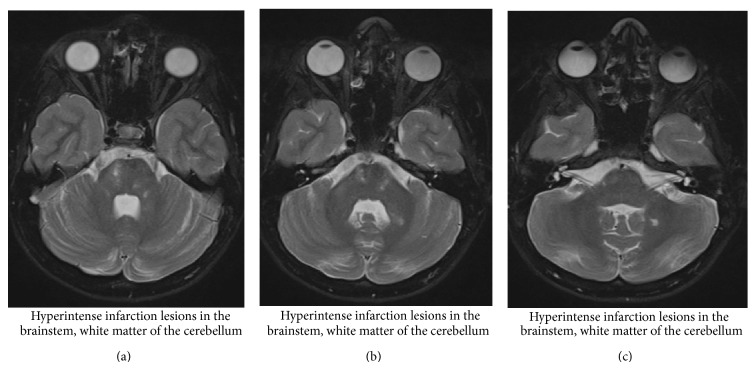
MRI (T2 axial images).

**Figure 3:**
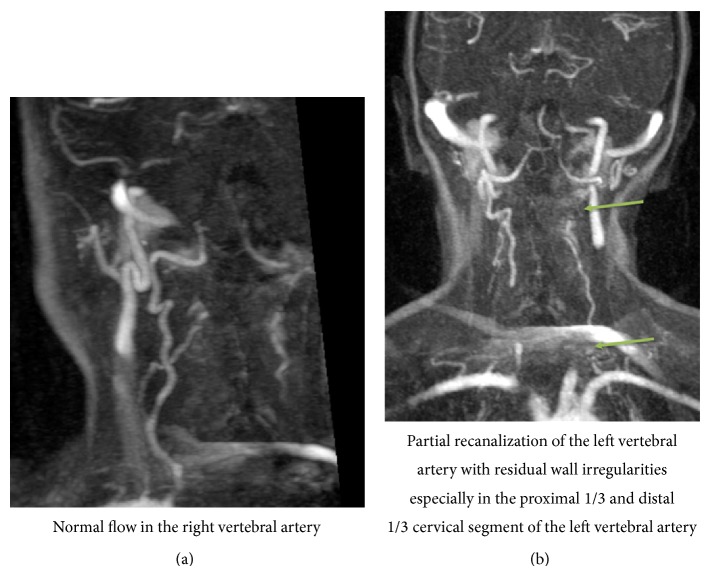
MRI angioscan (FLASH 3D coronal images after Gd-chelate administration).

The patient was transferred to the nearest university hospital, where immediate systemic thrombolysis with Activase (alteplase) (4 mg bolus injection and 36 mg over the following hour) was administered within four hours of the onset of the symptoms. The concurrent lack of clinical improvement led the clinicians to perform a catheter angiography in order to administer intra-arterial thrombolysis and an attempt to remove the thrombus. The procedure was performed under sedation with midazolam (Dormicum). Through a 5F H1 Headhunter catheter, selectively placed in the right vertebral artery, a Rebar 18 catheter is installed under guidance of a Traxcess 14 microwire into the thrombus at the tip of the basilar artery. The patient receives 2000 IE heparin intravenously and a total of 600.000 IE of urokinase are injected into the thrombus. The patency of the vessel initially improved, but the underlying dissection of the vessel made access more difficult and led to reclotting. After the procedure was completed, the result was similar to the situation prior to thrombolysis and clinical examination revealed a PedNIHSS of 19 with impaired consciousness (not being alert, requiring repeated stimulation to attend), left hemiparesis, bilateral clonus, hyperreflexia, and disability to communicate. A new CT scan excluded brain hemorrhage and, following a discussion between a child neurologist, an intensivist, an adult neurologist, and the interventional radiologist, heparinization was started and continued for 72 hours in order to attempt recanalization of the thrombus. Within 48 hours of admission, left hemiplegia and consciousness gradually improved and MRI (Figures 2(a)–2(c)) with angioscan (Figures 3(a) and 3(b)) indicated persistent thrombosis of the basilar artery with edema and ischemia of the right brain node. Finally, two weeks of LMWH treatment and subsequent oral warfarin (Sintrom, acenocoumarol) therapy resulted in a lasting improvement of the symptoms.

## 3. Outcome and Followup

Neurological evaluation four months after the event shows difficulties in coordination of his left arm, and evaluation in spontaneous use of his left arm (using Assisting Hand Assessment) is scored as 38%. The patient's gait is wide-based and he uses a wheelchair daily. Further his mother marks that he still has problems in concentrating and can be very emotional. His speak remained intact. MRI images on this moment still show occlusion of the cervical part of the left vertebral artery, no recanalization of the distal part of the basilar artery, and ischemic lesions in the pons and lacunar infarct zones in the left sight of the cerebellum.

One year on, the patient is still undergoing followup with child revalidation and neurology and hematology services and at his last consultation with the child hematologist there was still improvement in daily activity; he can dress himself and open a bottle himself and his gait is clearly improved. His warfarin therapy is continued.

## 4. Discussion

Vertebral artery dissection (VAD) after minor trauma is rare in children. VAD is further classified as being either traumatic or spontaneous. Spontaneous VAD lacks blunt or penetrating trauma as a precipitation factor and is caused by intrinsic factors that weaken the arterial wall. Between one and four percent of patients have a clear underlying connective tissue disorder such as Ehlers-Danlos syndrome type 4 and, more rarely, Marfan's syndrome. Genetic screening in our patient could not reveal any underlying connective tissue disorders, but he did have a history of trivial or minor injury, as many patients do with the so-called spontaneous VAD [1–3].

Acute basilar occlusion (ABO) as a complication of VAD is even more infrequent and its clinical presentation may manifest in a range of clinical symptoms, ranging from prodromal symptoms such as diplopia, dysarthria, vertigo, paresthesia, imbalance, and convulsive-like movements to gradual or sudden onset of severe motor and bulbar symptoms with impaired consciousness. This variety of clinical symptoms makes prompt diagnosis and treatment challenging imperative [4].

Image examinations are the most important tools for diagnosing VAD and ABO. Computed tomography (CT) scanning is usually the first imaging study performed to exclude hemorrhage in the brain, followed by spiral CT angiogram to identify occluded and dolichoectatic vessels. MRI and MR angiography are more sensitive than CT but limited because they frequently overestimate the degrees of stenosis. Catheter angiography is the gold standard and should be pursued as a first-line diagnostic test after CT scanning as soon as a decision is made to perform recanalization [5, 6].

There are no strict guidelines regarding the treatment of basilar artery occlusion in children [6–8]. Recommendations by AHA for the treatment of children with cervicocephalic arterial dissection are classes IIa and IIb (level of evidence C) recommendations which derive largely from adult series [9]. Either unfractionated heparin or low molecular weight heparin as a bridge to oral anticoagulation is recommended. Duration of treatment beyond 6 months is a reasonable option for children who develop recurrent symptoms or when there is radiographic evidence of a residual abnormality of the dissected artery. There have been a few reports on the use of tPA (tissue plasminogen activator) in children with ischemic stroke but safety and efficacy data for either intravenous or intra-arterial thrombolysis in children with acute arterial occlusion are lacking. The number of case reports in children reserving tPA within the time limit of 3 hours of stroke onset is limited. In the present case, we treated the patient with acute systemic thrombolysis followed by intra-artery thrombolysis and an attempt to remove the thrombus in an acute setting. Unfortunately, recanalization failed due to the fact that the underlying dissection of the vessel made access more difficult. Clinical improvement only was obtained after heparinization for 72 hours and lasting improvement was achieved after a two-week LMWH treatment and subsequent oral warfarin therapy. After each procedure or change in clinical response, a multidisciplinary team was involved in the next decision on further care and therapy. The rationale for pursuing our patient LMWH treatment on a long term basis is the extensive ischemic sequelae seen on his follow-up MRI images, the permanent occlusion of the left vertebral artery, and the tortuous tendency of both his left and right internal carotid arteries; these findings make a substantial higher risk of recurrent stroke [10].

## 5. Learning Points

In difficult procedures and precarious clinical cases such as these, we believe it is necessary to have more multidisciplinary discussion between emergency, critical care, interventional radiology, (child) neurology, and hematology physicians, from the moment a patient enters the hospital until the end of his follow-up therapy.
